# Irisin in Cardiovascular Health and Disease: A Review of Its Function as a Biomarker and Therapeutic Target in Atherosclerosis and Related Conditions

**DOI:** 10.3390/cimb48101020

**Published:** 2026-10-02

**Authors:** Siarhei A. Dabravolski, Sergey Shityakov, Nikita A. Mitkin, Ulyana V. Rozhkova, Stanislav A. Antonov, Aleksandra S. Utkina, Tatyana I. Kovyanova

**Affiliations:** 1 Department of Biotechnology Engineering, Braude Academic College of Engineering, Snunit 51, P.O. Box 78, Karmiel 2161002, Israel; sergedobrowolski@gmail.com; 2 Department of Biology and General Genetics, Sechenov First Moscow State Medical University ( Sechenov University), 119992 Moscow, Russia; 3 Institute for Atherosclerosis Research, Osennyaya Street 4-1-207, 121609 Moscow, Russia; 4 Laboratory of Molecular Genetic Modeling of Inflammaging, Institute of General Pathology and Pathophysiology, 8 Baltiyskaya Street, 125315 Moscow, Russia

**Keywords:** irisin, FNDC5, atherosclerosis, cardiovascular disease, myokine, biomarker, endothelial dysfunction, inflammation, exercise

## Abstract

Atherosclerotic cardiovascular disease (ASCVD) remains the leading cause of mortality worldwide, necessitating novel biomarkers and therapeutic targets. Irisin, a myokine derived from FNDC5, has emerged as a molecule of interest owing to its pleiotropic effects, yet its role in cardiovascular health is debated amid conflicting clinical findings and severe measurement challenges. This review synthesizes and critically evaluates the evidence on irisin in ASCVD, examining its molecular mechanisms, potential as a clinical biomarker, and hurdles for therapeutic application. Preclinical evidence has demonstrated that irisin exerts multifaceted cardioprotective effects: it mitigates cellular stress by enhancing mitochondrial quality control and inhibiting apoptosis and pyroptosis; preserves vascular integrity by promoting angiogenesis and preventing pathological remodeling; and enhances the efficacy of regenerative stem cell therapies. Most clinical studies have revealed an inverse correlation between circulating irisin levels and the presence and severity of ASCVD, heart failure, and related inflammatory conditions. However, these studies are predominantly observational and often cross-sectional, frequently with small sample sizes, and paradoxical elevations occur in certain metabolic states and during acute cardiac injury. Critically, the field is hampered by profound measurement inconsistencies, with reported values spanning picograms to micrograms per millilitre, making cross-study comparisons and clinical cutoffs currently impossible. Irisin appears to be an important endogenous factor in cardiovascular health, and preclinical evidence suggests that it is a promising candidate therapeutic target; however, clinical validation is lacking. Standardization of irisin assays remains the critical step required to determine whether irisin can become a reliable clinical biomarker.

## 1. Introduction

Cardiovascular diseases (CVDs) remain the foremost cause of mortality worldwide and are responsible for an estimated 20 million deaths annually, a figure projected to exceed 23 million by 2030 [1,2]. This immense clinical challenge is compounded by a substantial socioeconomic burden, stemming from the high costs of treatment and long-term care for patients with cardiovascular complications [3]. Established risk factors, such as dyslipidemia, smoking, and obesity, are well documented [4] and involve common pathophysiological pathways that promote atherosclerosis, the primary cause of most CVDs. Key among these mechanisms are endothelial dysfunction, which impairs vascular homeostasis, and a state of chronic, low-grade inflammation coupled with heightened oxidative stress, which collectively drive plaque formation and instability [5,6]. However, current therapeutic strategies targeting these processes often exhibit limited efficacy or are associated with significant adverse effects [7,8], underscoring the urgent need for novel, safe, and cost-effective strategies for both the prevention and treatment of CVD.

In this context, irisin has emerged as a molecule of significant interest. As discovered in 2012, irisin is a 112-amino acid myokine derived from the cleavage of its precursor, fibronectin type III domain-containing protein 5 (FNDC5), which is regulated by the transcriptional coactivator PGC-1α [9]. Although highly conserved between species, with nearly 100% homology between mouse and human variants, differences in the start codon of the FNDC5 gene raised initial concerns about the direct translatability of murine findings to human physiology [9,10]. While initially characterized as a product of skeletal muscle, subsequent research revealed a wider distribution, with cardiomyocytes identified as a primary source, alongside adipose tissue, the brain, and other organs [11].

Despite its discovery more than a decade ago, the physiological regulation and function of irisin remain the subject of intense debate. It was first identified as an exercise-induced hormone that promotes the “browning” of white adipose tissue (WAT), a metabolic effect with profound implications for energy expenditure [9]. This characterization, however, has been challenging. While exercise robustly induces WAT browning in some animal models [12,13], evidence for a similar response in humans remains equivocal, with many studies failing to confirm a significant exercise-induced rise in circulating irisin [14,15,16]. Factors such as exercise type, age, BMI, and metabolic status all appear to influence its serum concentration [17,18,19], but these inconsistencies have led researchers to question the primacy of exercise as its main stimulus. Accordingly, the present review treats exercise as the most extensively studied, but not definitively established, physiological modulator of irisin in humans.

These controversies extend beyond metabolism to the other reported pleiotropic effects of irisin. For example, while some studies suggest that irisin enhances cognitive function and synaptic plasticity by modulating brain-derived neurotrophic factor (BDNF) [20], these claims are tempered by the lack of a clear mechanism for its transport across the blood–brain barrier and the use of supraphysiological concentrations in experiments [21]. Similarly, its role in bone metabolism is conflicting, with reports suggesting that it promotes osteoblast differentiation [22,23], which is contradicted by findings in *FNDC5* knockout mice [24].

A significant shadow is cast over the field by profound methodological challenges, particularly in measurement. Early estimations of irisin levels via commercial ELISA kits were later shown to be unreliable because of antibody nonspecificity, yielding vastly different results from those of more rigorous quantitative mass spectrometry (MS) methods [9,25]. Although calibrated MS is now considered the “gold standard”, its repeatability at extremely low physiological concentrations of irisin (3–4 ng/mL in humans) remains a concern [26]. Furthermore, studies using *FNDC5* knockout mice further complicate the interpretation of findings; these animals often present no distinct phenotype under basal conditions but exhibit significant metabolic deficiencies when placed under physiological stress, such as a high-fat diet [13,24,27].

Given that cardiomyocytes are a major source of irisin and that the molecule has demonstrated protective effects against cardiac hypertrophy in preclinical models [28,29], its potential role in human cardiovascular health warrants closer examination.

However, the aforementioned controversies necessitate a critical evaluation of the literature. Therefore, this review aims to synthesize the current, often conflicting, evidence on the role of irisin in the pathophysiology of atherosclerotic cardiovascular disease (ASCVD). To achieve this goal, this study will first dissect the molecular mechanisms through which irisin affects cellular stress, vascular function, and key signaling pathways. Next, we will critically evaluate the clinical evidence positioning irisin as a potential biomarker, analyzing studies that link its circulating levels to various cardiovascular conditions. The pivotal role of exercise as a physiological modulator of irisin will then be examined. Finally, the review concludes by discussing the major gaps in our current understanding and proposing future research directions to clarify the therapeutic potential of irisin.

## 2. Study Design

### Literature Search

This work is a narrative review, not a systematic review. Its aim is to analyze and critically interpret the literature on irisin in cardiovascular disease, highlighting consensus, controversy, and gaps, rather than to exhaustively catalog all evidence. Formal systematic review requirements (exhaustive screening, predefined inclusion/exclusion criteria, risk-of-bias assessment) were therefore not applied. The literature was identified through searches of PubMed/MEDLINE, Scopus, and Web of Science (2012–2025) via combinations of “irisin,” “FNDC5,” “atherosclerosis,” “cardiovascular disease,” “myokine,” “biomarker,” “endothelial dysfunction,” “inflammation,” and “exercise,” with reference lists of retrieved articles also screened. Readers should interpret the evidence in light of the narrative design of this review.

## 3. Molecular Mechanisms of Cardioprotective Effects of Irisin

Preclinical studies using in vitro cell models and various animal models of CVD have begun to elucidate the complex molecular pathways through which irisin exerts its protective effects. While the primary focus of this review is ASCVD, findings from related cardiovascular and metabolic conditions are included where they illuminate shared mechanisms; these broader findings should be regarded as contextual and hypothesis-generating rather than direct ASCVD evidence. This evidence points toward irisin as a pleiotropic molecule that modulates fundamental cellular processes, including stress responses, vascular function, and intracellular signaling, to maintain cardiovascular homeostasis. Indeed, long-term systemic supplementation with irisin has been shown to be safe and effective, increasing the expression of cardioprotective genes while inhibiting those that are detrimental to heart physiology [30].

### 3.1. Attenuation of Cellular Stress and Maladaptive Remodeling

**Figure 1:**
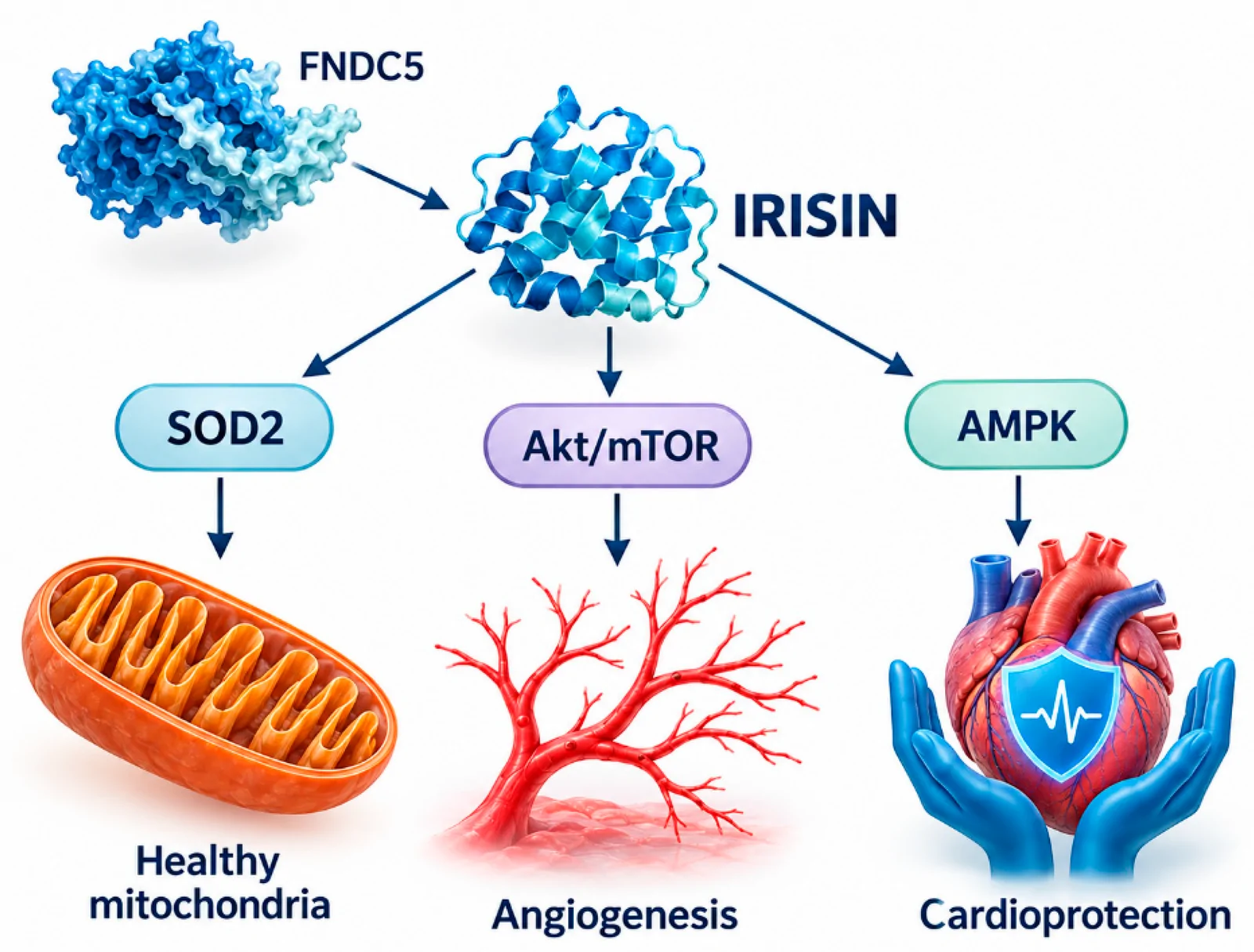
Schematic diagrams depicting the cardioprotective signaling pathways of irisin in ASCVD. Active irisin, cleaved from FNDC5, drives three protective axes: upregulating SOD2 to reduce oxidative stress and maintain healthy mitochondria; stimulating Akt/mTOR signaling to promote endothelial angiogenesis; and activating AMPK to provide direct myocardial cardioprotection against atherosclerotic disease progression.

A primary mechanism by which irisin confers cardioprotection involves mitigating cellular stress responses (Figure 1). In the context of myocardial ischemia/reperfusion (I/R) injury, irisin has profound protective effects, reducing infarct size and improving functional recovery [31,32]. This protection is closely linked to the preservation of mitochondrial function and the regulation of key signaling pathways. Mechanistically, irisin has been shown to increase the phosphorylation of p38 while suppressing key apoptotic markers such as active caspase 3 and cleaved PARP [31]. It also activates Opa1-induced mitophagy to clear damaged mitochondria [33], upregulates the mitochondrial ubiquitin ligase MITOL to negate ER stress [34], and enhances the activity of mitochondrial antioxidants such as SOD2 (Figure 1) [32]. This robust antiapoptotic effect is further mediated by the AMPK pathway, which is critical for protecting cardiomyocytes (Figure 1) from H/R-induced ER stress [35] and is especially vital in diabetic models of I/R injury [36].

In addition to I/R, irisin counters a range of pathological insults. In models of pressure overload-induced cardiac hypertrophy, FNDC5/irisin clearly has a protective effect. *FNDC5* ablation aggravates hypertrophic damage, whereas its overexpression or supplementation attenuates it, an effect mediated by the induction of protective autophagy via the AMPK-ULK1 signaling pathway [27]. This finding is complemented by findings that irisin, which is cleaved from FNDC5 in an ADAM10-dependent manner, improves cardiac function by regulating the AMPK-mTOR axis [29]. In models of doxorubicin-induced cardiotoxicity, it alleviates oxidative stress via the Akt/GSK3β/Fyn/Nrf2 axis [37] and reverses perivascular fibrosis by targeting UCP2 to block the endothelial-to-mesenchymal transition (EndMT) [38]. Furthermore, in response to H_2_O_2_-induced oxidative stress, irisin enhances cardiomyocyte proliferation by upregulating *miR-19b*, which in turn reactivates the prosurvival Akt/mTOR pathway [39]. However, notably, the effects of irisin may be dose dependent, as one study reported that excessive irisin expression could paradoxically increase ROS production and apoptosis in cardiomyocytes under hypoxic conditions, suggesting that a therapeutic window exists [40].

### 3.2. Promotion of Vascular Health and Integrity

The protective effects of irisin extend directly to the vasculature. It directly rescues oxLDL-impaired angiogenesis (Figure 1) via the Akt/mTOR/Nrf2 pathway [41] and stimulates angiogenesis post-MI through ERK signaling [42]. A key mechanism through which irisin prevents vascular damage is the inhibition of EndMT. In diabetic models, irisin alleviates cardiac fibrosis by suppressing high glucose-induced EndMT via the modulation of Smad2/3 and Smad7 signaling [43]. Irisin also actively counteracts atherogenesis by suppressing the expression of endothelial adhesion molecules [44] and reversing nicotine-induced vascular thickening [45]. Moreover, it protects against vascular calcification by activating the AMPK/Drp1 pathway to inhibit the osteoblastic transformation of vascular smooth muscle cells [46].

### 3.3. Central Role of Key Intracellular Signaling Hubs

The diverse protective effects of irisin converge on a few critical intracellular signaling hubs. The AMPK pathway is a primary target that protects against hypertrophy [27,28] and I/R injury [35]. The Akt/PI3K pathway is also central, but its role appears to be highly context dependent. In some settings, irisin activates Akt signaling to promote survival, such as against H_2_O_2_-induced stress [39]. In stark contrast, in a rat model of pressure overload (TAC), irisin treatment improved cardiac remodeling by inhibiting the Akt/mTOR signaling pathway, suggesting that it can fine-tune this axis depending on the specific pathological stimulus [47]. The initiation of these signals is increasingly understood to occur via the binding of irisin to integrin receptors, particularly αVβ5, a process that can be allosterically enhanced by extracellular Hsp90α [48].

### 3.4. Enhancement of Regenerative Therapies

A novel and promising area of research is the role of irisin in enhancing cell-based regenerative therapies for cardiac repair. Pretreatment with irisin has been shown to improve the therapeutic efficacy of various stem cell populations. In models of MI, *FNDC5* overexpression or irisin treatment improved the survival and paracrine effects of transplanted bone marrow-derived mesenchymal stem cells (BM-MSCs), partly by increasing their secretion of proangiogenic exosomes [49,50]. Similarly, it enhances the survival and neovascularisation capacity of transplanted cardiac progenitor cells (CPCs) [51] and increases the cardiac homing of adipose-derived MSCs (ADSCs) to the ischemic heart [52].

### 3.5. Regulation of Irisin Expression and Action

Understanding the factors that regulate irisin itself is crucial. The sympathetic nervous system appears to play a key role, as demonstrated in zebrafish, where the cardiostimulatory effects of irisin are attenuated by the β-adrenergic blocker propranolol, indicating the involvement of the β-adrenergic pathway in mediating its cardiovascular effects [53]. Hypoxia is another critical regulator, although its effects appear complex and context dependent. Chronic systemic hypoxia in mice leads to muscle atrophy and a decrease in *FNDC5* expression, a process negatively regulated by HIF1α [54]. In stark contrast, direct exposure of cardiomyocyte cell lines to hypoxia resulted in an increase in *FNDC5*/irisin expression [55,56], suggesting a local protective response in the heart that is distinct from the systemic response.

Irisin levels can also be modulated by other signaling pathways and pharmacological agents. For example, activation of the farnesoid X receptor (FXR), a nuclear receptor involved in bile acid metabolism, has been shown to be a direct transcriptional activator of *FNDC5*, leading to improved hyperlipidemia and reduced atherosclerosis in *ApoE*^−/−^ mice [57]. Additionally, vasodilatory drugs used to treat myocardial ischemia, such as iloprost and sildenafil, have been found to restore irisin levels that are depleted following MI, suggesting that improving tissue perfusion may also support endogenous irisin production [58].

In conclusion, collective preclinical evidence has demonstrated that irisin is a remarkably pleiotropic molecule with a multipronged protective effect on the cardiovascular system. It acts as a potent cellular guardian, shielding cardiomyocytes and vascular cells from a wide array of pathological insults, from ischemia and oxidative stress to drug-induced toxicity, by promoting mitochondrial quality control and inhibiting programmed cell death (Table 1). Concurrently, it actively preserves vascular health by inhibiting key processes in atherogenesis, such as endothelial dysfunction, inflammation, and pathological cellular transitions, while promoting angiogenesis. These diverse effects are orchestrated through a core set of signaling hubs, primarily the AMPK and AKT pathways, initiated by binding to integrin receptors. Furthermore, irisin shows significant promise as an adjuvant for regenerative medicine, enhancing the efficacy of stem cell therapies. The expression and action of irisin are themselves subject to a complex regulatory network involving autonomic signals, local oxygen tension, and other systemic pathways, which underscores its deep integration into cardiovascular physiology.

## 4. Irisin as a Clinical Biomarker in Cardiovascular Disease

Building on mechanistic insights from preclinical models, a substantial body of clinical research has investigated the associations between circulating irisin levels and the presence, severity, and prognosis of ASCVD in human populations. These studies, which were conducted across diverse patient cohorts and clinical settings, collectively explore the potential of irisin as a valuable biomarker. For clarity, the evidence presented below is organized to distinguish ASCVD-specific findings (coronary artery disease, myocardial infarction, stroke, atherosclerosis, and aortic stenosis) from broader cardiovascular and metabolic associations (heart failure, type 2 diabetes, metabolic syndrome, thyroid dysfunction, and inflammatory disorders), which are presented as supportive and contextual rather than as primary ASCVD evidence.

Notably, the wide variation in reported concentrations and units across studies renders direct comparisons of absolute values invalid and precludes the establishment of any clinical cutoff. The findings summarized below should therefore be interpreted qualitatively rather than quantitatively, and the numerical values presented in Table 2 are not clinically applicable.

### 4.1. The Predominant Finding: An Inverse Association with Cardiovascular Risk and Disease Severity

The most consistent finding across a large volume of clinical research is that lower circulating irisin levels are generally associated with an increased prevalence and severity of ASCVD. However, most of these studies are observational and frequently cross-sectional, and many are limited by small sample sizes and heterogeneous assay methods.

In patients with stable coronary artery disease (CAD), serum irisin is inversely correlated with the SYNTAX score, a measure of coronary lesion complexity [59], and with the severity of stenosis [60]. It is also lower in patients with a diagonal earlobe crease (DELC), a curious physical marker of CAD [62]. This is supported by studies showing significantly lower irisin levels in patients with either stable CAD or acute MI than in healthy controls [61]. Similarly, lower irisin is linked to coronary artery ectasia [64], a greater burden of coronary artery calcification [63], and an increased likelihood of AMI [68,69]. This phenomenon extends to other structural heart diseases, such as severe aortic stenosis, where lower plasma irisin is negatively associated with the severity of aortic valve calcification [65]. In obese populations, as demonstrated in a study where patients with carotid atherosclerosis had significantly lower irisin levels (28.05 ± 8.33 μg/mL) than normal-weight controls did (63.38 ± 10.27 μg/mL) [67], these absolute values should be interpreted with caution given the heterogeneity of the assay. Lower irisin levels are associated with subclinical atheroma plaques [66], whereas higher levels are linked to a better profile with fewer cardiovascular risk factors [91]. This association with adverse outcomes is also observed in acute ischemic stroke, where low irisin levels predict poor early functional outcomes [70]. This extends to comorbidities, with lower irisin levels also found in CHD patients suffering from depression [87].

This inverse relationship is particularly pronounced in patients with metabolic and inflammatory diseases. In individuals with type 2 diabetes (T2DM), lower irisin is associated with atherosclerosis [73], macrovascular complications [74], the presence of chronic total occlusions (CTO) [77], and an increased risk of CVD in general [75]. It is also correlated with the accumulation of advanced glycation end products (AGEs), a key driver of diabetic vascular damage [76]. The link to heart failure (HF) is also strong, with lower irisin levels observed in patients with chronic HF, where they correlate with systemic inflammation (CRP) and functional decline [83], and in Egyptian patients with HF, both with and without MI [79]. Specifically, in T2DM patients, lower irisin levels predict the presence of HF [80] and future adverse events [94]. Conversely, higher baseline irisin levels in HFrEF patients predict a future improvement in the ejection fraction [81]. Similar trends are observed in chronic inflammatory conditions such as axial spondyloarthritis, where low irisin is linked to subclinical atherosclerosis [84], and in severe hypothyroidism, where low irisin is associated with myocardial injury [85]. However, this is not universal, as no associations were found between irisin and atherosclerosis markers in patients with subclinical hypothyroidism [86], and another study in T2DM patients revealed no significant difference in absolute irisin levels compared with those in controls, despite observing the expected inverse correlations with risk markers, such as BMI and CRP [78].

### 4.2. Context-Dependent and Paradoxical Associations

While the inverse association is predominant, the clinical picture is heterogeneous and at times contradictory. Some studies report context-dependent or even paradoxical elevations in irisin levels, suggesting that its role as a biomarker may be modulated by a specific pathological state. These contradictory findings underscore that irisin cannot yet be regarded as a robust or universally reliable biomarker.

For example, some cohorts of patients with metabolic syndrome (MetS) [88] or MetS with androgenetic alopecia [95] show paradoxically higher irisin levels, which may suggest an initial compensatory response to metabolic stress. This is also observed in children with obesity, where one study reported higher irisin values (187.96 ng/mL) than in controls (169.73 ng/mL), with levels correlating positively with adiposity and markers of insulin resistance [89]. These absolute values are assay-dependent and not directly comparable with those of other studies.

In another large study of Chinese children, while irisin levels were not directly related to physical activity, they were negatively correlated with BMI and blood pressure only in the inactive subgroup, suggesting a complex interplay between irisin and lifestyle factors [90].

Furthermore, the clinical subtype of a disease can dictate the behavior of irisin. In HF, while lower levels are common, one study reported that patients with preserved ejection fraction (HFpEF) had significantly higher irisin levels than those with reduced ejection fraction (HFrEF) [82]. The timing of measurement is also critical in acute settings. One study in post-STEMI patients revealed that the highest quartile of irisin at admission was associated with a greater risk of adverse events [71], whereas other studies linked lower irisin levels to worse outcomes [72]. This discrepancy supports the hypothesis that a surge in irisin release from massive myocardial injury may reflect the extent of damage rather than the baseline protective capacity.

### 4.3. The Role of FNDC5 Gene Polymorphisms

Investigations into the genetic basis for irisin variability have yielded inconsistent results across different ethnic populations. In an Egyptian population, the G allele of rs3480 and the A allele of rs726344 were identified as significant risk factors for myocardial infarction [92]. However, a study in a Turkish AMI population revealed no significant associations for the same SNPs, although a specific haplotype (GGT) appeared protective [68]. Research in a Chinese cohort revealed no link between rs16835198 and rs3480 and susceptibility to CAD or hypertension [93]. These inconsistent findings across Egyptian, Turkish, and Chinese cohorts remain unresolved. Whether they reflect genuine population-specific effects, differences in study design and sample size, or simply stochastic variation cannot be determined from the available data, and no conclusion about the role of FNDC5 polymorphisms in ASCVD susceptibility can currently be drawn.

**Figure 2:**
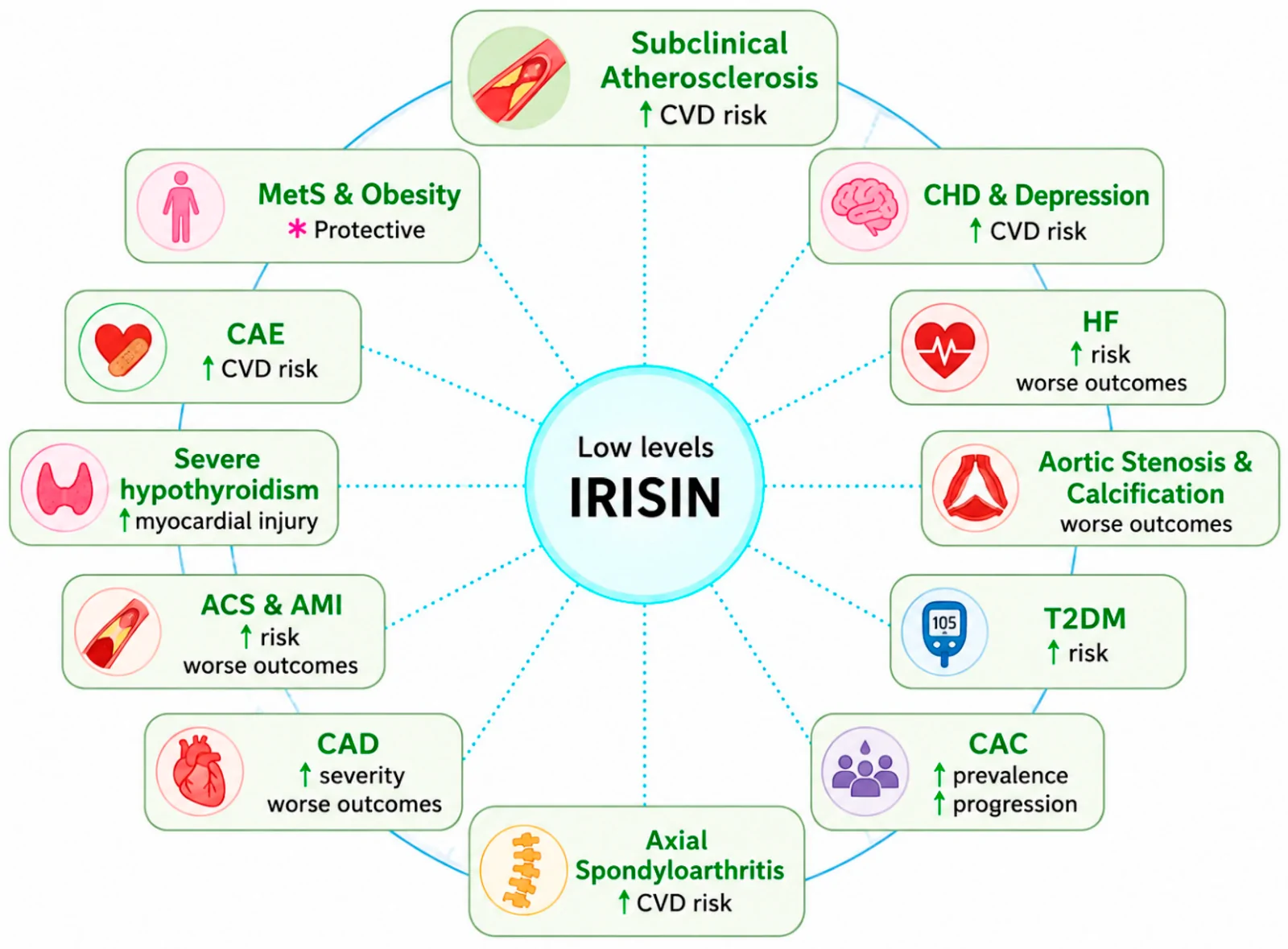
Associations of low circulating irisin levels with cardiovascular and related conditions. Upward arrows (↑) indicate increased risk, greater severity, or worse outcomes. The magenta asterisk (*) denotes contradictory findings in which higher (rather than lower) irisin levels have been reported (MetS and obesity).

In conclusion, the clinical evidence paints a compelling, albeit complex, picture of irisin’s role as a cardiovascular biomarker (Figure 2). The predominant trend across a wide spectrum of diseases, from stable CAD to heart failure and chronic inflammatory states, is an inverse relationship: lower circulating irisin is often associated with greater disease burden and poorer outcomes. However, given the observational and frequently cross-sectional design of most studies, the small sample sizes of many cohorts, and the substantial heterogeneity and contradictory findings, these results should be interpreted as hypothesis-generating. Irisin may be a candidate biomarker of cardiovascular health, but it has not yet been validated for clinical use.

On the other hand, this interpretation is challenged by paradoxical findings in certain metabolic states and acute settings, suggesting that irisin levels may reflect different processes, such as chronic deficiency versus acute injury or compensatory response, depending on the clinical context (Table 2). Furthermore, the lack of consistent findings in genetic studies indicates that environmental and lifestyle factors likely play a more dominant role in regulating the impact of irisin. Ultimately, while irisin holds immense promise, its full potential as a reliable biomarker can be realized only after the critical issue of measurement standardization is resolved. Until then, no proposed cutoff value should be regarded as clinically actionable.

## 5. The Role of Exercise as a Key Physiological Modulator of Irisin

Exercise is the most extensively studied physiological modulator of irisin production, providing a crucial bridge between its mechanistic functions and its clinical relevance in cardiovascular health. However, as noted in the Introduction, the human evidence remains heterogeneous, and the exercise–irisin relationship should not be regarded as definitively established. The investigation of exercise-induced irisin reveals how lifestyle interventions can directly influence this protective myokine, offering a nonpharmacological avenue for enhancing cardiovascular resilience.

Research in preclinical models has clearly established that physical activity can robustly upregulate *FNDC5*/irisin expression, leading to tangible functional benefits. In a mouse model of radiation-induced heart disease (RIHD), a condition that impairs cardiac function, exercise improved aerobic fitness and cardiac performance. This effect was accompanied by elevated *FNDC5*/irisin expression and the selective activation of mitophagy, suggesting that exercise can prevent the development of RIHD, at least in part, through an irisin-mediated mitochondrial quality control pathway [96]. Similarly, in a mouse model of myocardial infarction, various forms of exercise, including aerobic and resistance training, increased myocardial irisin expression, which was linked to activation of the PINK1/Parkin mitophagy pathway, reduced oxidative stress, and improved cardiac function [97]. In atherosclerotic *ApoE*^−/−^ mice, both high-intensity interval training (HIIT) and moderate-intensity continuous training (MICT) promote the expression of irisin, which is associated with reduced oxidative damage and improved lipid profiles [98].

These preclinical findings are strongly supported by evidence from both animal and human studies linking exercise-induced irisin to improved cardiovascular parameters. In rats subjected to 8 weeks of training prior to induced AMI, higher postexercise serum irisin levels were strongly negatively correlated with markers of cardiac injury (CK-MB, troponin), ECG abnormalities (ST elevation, QTc prolongation), and fibrosis (collagen deposition, caspase-3) but positively correlated with total antioxidant status [99]. In another study, a 12-week exercise programme in rats led to increased circulating irisin levels, which positively correlated with improved cardiac function, including increased ejection fraction and fractional shortening [100]. The causal link between irisin signaling and the benefits of exercise was demonstrated in diabetic rats, where the cardioprotective effects of treadmill training were significantly diminished when the irisin receptor (αVβ5) was blocked [101]. This crucial experiment confirmed that irisin signaling is functionally required for at least part of the cardioprotective effect of exercise.

Furthermore, studies in human populations directly link cardiorespiratory fitness with irisin and better cardiometabolic health. In a large study of healthy adults spanning a wide age range, higher cardiorespiratory fitness (measured as VO_2_peak) was directly proportional to higher serum irisin levels. In turn, higher irisin levels are significantly correlated with a better cardiometabolic profile, including lower body fat, blood pressure, fasting glucose, and triglyceride levels and higher HDL cholesterol levels [102]. This holds true even in at-risk populations; in severely obese patients, a 6-week program of moderate-intensity aerobic training led to a significant increase in serum irisin, which was accompanied by improved cardiorespiratory capacity and a better inflammatory and oxidative stress profile [103]. In a post-MI rehabilitation context, while exercise was superior in improving myocardial contractility, the use of dihydromyricetin to mimic exercise’s effect on irisin also successfully increased its serum levels, highlighting the potential for exercise mimetics to target this pathway [104].

In summary, exercise is the most extensively investigated physiological modulator of endogenous irisin levels. Preclinical evidence consistently shows that physical activity stimulates irisin production, which in turn activates protective cellular pathways, reduces cardiovascular risk factors, and improves overall cardiac function. In humans, the picture is more heterogeneous: while some studies link higher cardiorespiratory fitness to higher irisin and better cardiometabolic health, others fail to detect a significant exercise-induced rise in circulating irisin. The findings linking irisin directly to cardiorespiratory fitness in humans and the demonstration that blocking its receptor negates many of exercise’s benefits in animal models suggest that irisin may be a key mediator of exercise-induced cardioprotection, although this requires further validation in human studies. This makes the exercise–irisin axis a promising focus for both preventive health strategies and the development of novel therapeutic approaches.

## 6. Discussion and Future Directions

The discovery of irisin over a decade ago sparked considerable interest in its potential as a therapeutic target for metabolic and cardiovascular diseases. This review analyzes a large and complex body of literature revealing that irisin is a pleiotropic molecule with significant cardioprotective properties. The evidence, spanning from molecular mechanisms in preclinical models to epidemiological and biomarker studies in diverse human populations, consistently points toward a beneficial role for irisin in maintaining cardiovascular homeostasis. However, the field is also marked by significant controversies and methodological challenges that require critical discussion to contextualize the findings and guide future research effectively.

### 6.1. Irisin’s Cardioprotective Role

The cumulative evidence highlights a compelling picture of irisin as a multifaceted guardian of the cardiovascular system. At the molecular level, irisin is not limited to a single mechanism but orchestrates a coordinated defense against cellular stressors. It enhances cell survival by promoting protective autophagy and mitophagy while simultaneously inhibiting multiple pathways of programmed cell death, including apoptosis and inflammatory pyroptosis. Its ability to activate central signaling hubs such as AMPK and Akt, primarily through integrin receptors, allows it to suppress oxidative stress, reduce inflammation, and preserve mitochondrial function. These cellular benefits translate directly into improved vascular health, where irisin promotes angiogenesis, inhibits pathological processes such as endothelial-to-mesenchymal transition and vascular calcification, and counteracts the foundational steps of atherogenesis. Furthermore, the novel finding that irisin can increase the efficacy of stem cell-based regenerative therapies opens an exciting new frontier for its application.

This mechanistic foundation is strongly supported by clinical evidence. The predominant finding across dozens of human studies is a robust inverse correlation between circulating irisin levels and the prevalence and severity of ASCVD and its associated conditions, from stable coronary artery disease and aortic stenosis to chronic inflammatory states such as axial spondyloarthritis. A lower irisin level is frequently linked to a greater burden of disease, worse outcomes after acute ischemic events, and an increased risk of complications, particularly in patients with T2DM. However, these associations are derived largely from observational and cross-sectional studies, and causality cannot be inferred.

The role of exercise as a widely studied physiological modulator of irisin secretion, with higher cardiorespiratory fitness often correlated with higher irisin levels and better cardiometabolic health, supports, but does not yet confirm, this link. Given the heterogeneity of human data, physical activity should be regarded as a promising, rather than established, means of harnessing this innate protective system.

### 6.2. Addressing Key Controversies and Methodological Hurdles

Despite compelling evidence, the field remains hampered by controversies and methodological issues that must be addressed. A fundamental barrier to synthesizing clinical data is the profound inconsistency in irisin measurement. As highlighted in the introduction and starkly illustrated in Table 3, reported circulating irisin concentrations vary by several orders of magnitude, making direct comparisons of absolute values between studies almost meaningless. Consequently, the numerical cutoffs listed in these tables are presented for illustrative purposes only and should not be interpreted as clinically applicable thresholds.

The extreme variance shown in Table 3, from picograms to micrograms per milliliter, stems directly from the use of different commercial ELISA kits with varying specificities versus more rigorous but less accessible mass spectrometry methods [25]. This “measurement crisis” is arguably the single greatest obstacle preventing the translation of irisin into a reliable clinical tool.

Beyond this central issue, this review has highlighted apparently contradictory findings. While low irisin is generally associated with poor outcomes, some studies report paradoxical elevations in specific contexts, such as in children with obesity, adults with metabolic syndrome, or during acute STEMI [71]. These findings do not necessarily support the protective role of irisin. Instead, they likely reflect the complexity of its biology. An initial compensatory rise in response to metabolic stress may occur before a state of “irisin resistance” or eventual depletion ensues. Similarly, an acute surge following massive tissue injury may be a marker of damage severity rather than a baseline protective capacity. The inconsistent results from genetic studies on *FNDC5* polymorphisms further underscore this complexity, suggesting that genetic predisposition is likely modulated by strong gene-environment interactions.

### 6.3. Gaps in Knowledge and Future Research Directions

To move the field forward and translate the promise of irisin into clinical reality, several key gaps in our knowledge must be addressed. Future research should prioritize the following areas.

#### 6.3.1. Standardization of Measurement

The development and widespread adoption of a validated, international standard for irisin quantification, preferably based on mass spectrometry, is paramount. This is the single most important step needed to harmonize clinical data and establish reliable diagnostic and prognostic thresholds.

#### 6.3.2. Longitudinal Human

Studies: The majority of clinical studies are cross-sectional. There is an urgent need for large-scale, prospective longitudinal studies that track irisin levels over time—using a standardized assay, in initially healthy cohorts. This will be crucial for determining whether low irisin is a true predictor of future cardiovascular events rather than just a marker of existing disease.

#### 6.3.3. Clinical Trials of Recombinant Irisin

The wealth of preclinical evidence provides a rationale for testing the therapeutic efficacy of recombinant human irisin, although no such trials have yet been conducted, and its therapeutic efficacy in humans remains unproven. Well-designed, randomized controlled trials are needed to assess its safety and effectiveness in specific patient populations, such as those recovering from MI, those undergoing cardiac rehabilitation, and those with stable HF.

#### 6.3.4. Deepening Mechanistic Understanding

While we have identified key pathways such as the AMPK pathway, further research is needed to delineate the complete irisin-receptor signaling axis in different cardiovascular cell types and to understand the context-dependent regulation of its downstream effectors. Elucidating the mechanisms behind its paradoxical regulation in certain disease states and its interactions with other myokines and hormones will be crucial for targeted therapeutic development.

## 7. Conclusions

Irisin has emerged from over a decade of research as a promising pleiotropic molecule with a potential role in cardiovascular health. Preclinical evidence suggests that irisin functions as an endogenous cardioprotective factor, acting through a network of key molecular pathways to combat cellular stress, preserve vascular integrity, and reduce inflammation. Clinically, lower circulating irisin is frequently associated with increased burden and severity of atherosclerotic cardiovascular disease, suggesting that it may be a candidate biomarker. The ASCVD-specific evidence is the most consistent, whereas associations with heart failure, T2DM, and other conditions remain supportive and require further validation.

However, irisin currently remains an investigational biomarker, with no established reference range, no validated diagnostic or prognostic threshold, and no demonstrated therapeutic efficacy in humans. Exercise remains the most extensively investigated physiological stimulus for irisin, although human data are heterogeneous, and the exercise–irisin relationship has not yet been definitively established. While significant methodological challenges and biological complexities remain, convergent findings from preclinical and clinical research underscore the importance of the irisin system as a promising target for future diagnostic and therapeutic strategies aimed at mitigating the global impact of cardiovascular disease [105].

## Figures and Tables

**Table 1:** Summary of preclinical evidence for the cardioprotective mechanisms of irisin.

Biological Process	Model/System	Effect of Irisin	Key Molecular Pathway(s) Modulated	Observed Outcome	References
I/R Injury & Apoptosis	Langendorff rat heart (I/R)	↓ Infarct Size	↑ p38 phosphorylation; ↓ Caspase-3	Improved functional recovery, protected mitochondria	[31]
	MI/R hearts	↓ Oxidative Stress	↑ SOD2 mitochondrial localization	Reduced I/R-induced apoptosis	[32]
	Mouse hearts (post-MI)	↓ Apoptosis	↑ Opa1-induced mitophagy	Protected cardiomyocytes from ischemic injury	[34]
	H9c2 cells (H/R)		↑ AMPK; ↓ ER Stress (GRP78, CHOP)	Reduced ER stress-induced apoptosis	[35]
	Diabetic mice (MI/R)	↓ Injury	↑ AMPK pathway	Attenuated MI/R injury, improved mitochondrial function	[36]
	MI/R hearts/H9c2 cells	↓ ER Stress	↑ MITOL	Protected against MI/R injury by negating ER stress	[34]
Hypertrophy	Mouse hearts (TAC)	↓ Hypertrophy	↑ AMPK → ↑ ULK1	Induced protective autophagy	[27]
			↑ AMPK; ↓ mTOR (ADAM10-dependent)	Improved cardiac function & attenuated fibrosis	[28]
	Rat hearts (TAC)	↓ Oxidative Stress	↓ Akt/mTOR/GSK3β	Improved cardiac remodeling	[47]
Drug/Toxin-induced Injury	H9c2 cells (Doxorubicin)	↓ Apoptosis	↑ Akt/GSK3β/Fyn/Nrf2	Alleviated cardiotoxicity and oxidative stress	[37]
Oxidative Stress	H9c2 cells (H_2_O_2_)	↑ Viability	↑ miR-19b → ↓ PTEN → ↑ Akt/mTOR	Enhanced proliferation, prevented apoptosis	[39]
	Mouse hearts (hypoxia)	↔ ↑ Apoptosis	↑ ROS production	Excessive irisin caused mitochondrial overdrive	[40]
CPCs	MI mouse hearts	↑ Repair	↑ Proliferation, ↓ Apoptosis	Enhanced CPC-induced cardiac regeneration	[51]
MSCs	MI mouse hearts (BM-MSCs)	↑ Survival	↑ FNDC5 overexpression	Improved MSC engraftment and paracrine effects	[49]
		↑ Angiogenesis	↑ Exosome secretion (VEGF, bFGF)	Improved paracrine effects & cardiac function	[50]
	MI/R mice (ADSCs)	↑ Homing	↑ CSF2/CSF2RB signaling	Increased therapeutic efficacy of IV-delivered ADSCs	[52]

Legend: ↑: Increase/activation. ↓: Decrease/inhibition. ↔: Context-dependent effects. →: Leads to/induces. ADSC: adipose-derived stem cell; AngII: angiotensin II; BM-MSCs: bone marrow-derived mesenchymal stem cells; CKD: chronic kidney disease; CPC: cardiac progenitor cell; DOX: doxorubicin; EndMT: endothelial-to-mesenchymal transition; FXR: farnesoid X receptor; H/R: hypoxia/reoxygenation; I/R: ischemia/reperfusion; LPS: lipopolysaccharide; MSCs: mesenchymal stem cells; MI: myocardial infarction; TAC: transverse aortic contraction.

**Table 2:** Summary of clinical studies on circulating irisin as a biomarker in cardiovascular disease and related conditions.

Condition/Patient Cohort	Population	Irisin’s Role as a Biomarker	Key Quantitative Findings & Cutoffs	References
Coronary Artery Disease (CAD) & Atherosclerosis				
Stable CAD & Severity	63 Turkish elderly	Low levels = Worse outcome	High SYNTAX: 127.9 vs. Low SYNTAX: 224.7 ng/mL.	[59]
	62 Pakistani middle-aged	Low levels = Worse outcome	Lower in severe CAD (9.3 µg/mL) vs. mild CAD (15.3 µg/mL).	[60]
	71 Greek patients	Low levels = Risk factor	Lower in stable CAD (161.2 ng/mL) and MI (143.5 ng/mL) vs. controls (217.2 ng/mL).	[61]
	45 Chinese elderly	Low levels = Risk factor	Lower in CAD (175.0 µg/L) & DELC group vs. controls.	[62]
Coronary Artery Calcification (CAC)	1038 Japanese men	High levels = Protective	Higher irisin associated with lower CAC prevalence and progression.	[63]
Coronary Artery Ectasia (CAE)	50 Turkish middle-aged	Low levels = Risk factor	Lower in CAE (1.85 pg/mL) vs. controls (5.78 pg/mL).	[64]
Aortic Stenosis & Calcification	293 Chinese elderly	Low levels = Worse outcome	Negatively associated with aortic valve calcification severity.	[65]
Subclinical Atherosclerosis (Obesity)	61 Spanish middle-aged	Low levels = Risk factor	Lower in subjects with atheroma (15.4 ng/mL) vs. without (20.9 ng/mL).	[66]
Obesity with Carotid Atherosclerosis	156 Chinese elderly	Low levels = Risk factor	Lower in obese CAS (28.05 µg/mL) vs. controls (63.38 µg/mL).	[67]
Acute Coronary Syndromes (ACS) & Stroke				
Acute Myocardial Infarction (AMI)	225 Turkish elderly	Low levels = Risk factor	Lower in AMI (3.14 ng/mL) vs. controls (4.02 ng/mL); levels drop post-AMI.	[68]
	44 Turkish elderly (STEMI)	Low levels = Risk factor	Lower in STEMI (4.95 ng/mL) vs. controls (12.5 ng/mL). Cutoff: <6.71 ng/mL.	[69]
Acute Ischemic Stroke (AIS)	324 Chinese elderly	Low levels = Worse outcome	Lower in poor outcome group (257.1 ng/mL) vs. good outcome (315.5 ng/mL).	[70]
Prognosis post-PCI for AMI	207 Chinese elderly	Low levels = Worse outcome	Lower in MACE group (49.35 ng/mL) vs. no-MACE (78.93 ng/mL).	[71]
Prognosis post-STEMI	399 Taiwanese middle-aged	Contradictory/High levels = Worse	Highest quartile (>0.60 μg/mL) had ~4-fold increased risk of adverse events.	[72]
Type 2 Diabetes (T2DM) & Cardiometabolic Risk				
T2DM with Atherosclerosis	69 Egyptian middle-aged	Low levels = Risk factor	Lower in T2DM + AS (32.9 pg/mL) vs. controls (473.6 pg/mL).	[73]
T2DM & Macrovascular Complications	138 Saudi middle-aged	Low levels = Risk factor	Lower in T2DM + MVC (0.50 µg/mL) vs. without (0.58 µg/mL).	[74]
T2DM & CVD Risk	135 Egyptian middle-aged	Low levels = Risk factor	Lower in CVD group (55.77 ng/mL) vs. non-CVD (115.5 ng/mL).	[75]
T2DM & AGEs Accumulation	362 Chinese middle-aged	Low levels = Marker of damage	Lower in T2DM (16.24 ng/mL) vs. controls (24.35 ng/mL).	[76]
T2DM & CTO	86 Turkish elderly	Low levels = Risk factor	Lower in patients with diabetes, but not correlated with SYNTAX score.	[77]
T2DM (General)	65 Egyptian middle-aged	Context-dependent	No difference vs. controls, but inverse correlation with BMI, CRP, LDL.	[78]
Heart Failure (HF)				
HF with or without MI	66 Egyptian middle-aged	Low levels = Risk factor	Lower in HF groups (48.7–54.3 ng/mL) vs. controls (73.1 ng/mL).	[79]
T2DM & HF Presence/Prognosis	226/183 Ukrainian patients	Low levels = Risk factor	Lower irisin predicts HF presence and progression to HFrEF.	[80]
Prediction of HF Improvement	313 Ukrainian elderly HFrEF	High levels = Favorable outcome	Higher in HFimpEF group (8.23 ng/mL) vs. HFrEF (4.37 ng/mL).	[81]
HFpEF vs. HFrEF	29 Italian elderly	Contradictory/High levels in HFpEF	Higher in HFpEF (7.72 ng/mL) vs. HFrEF (2.77 ng/mL).	[82]
Chronic HF & Function	292 Pakistani elderly	Low levels = Worse outcome	Lower in CHF (224–229 pg/dL) vs. controls; correlated with LVEF and CRP.	[83]
Other Conditions & Context-Dependent Findings				
Axial Spondyloarthritis (axSpA)	725 Spanish middle-aged	Low levels = Risk factor	Lower in patients with sacroiliitis (2.12 ng/mL) vs. without (2.60 ng/mL).	[84]
Severe Hypothyroidism	25 Chinese middle-aged	Low levels = Marker of injury	Lower in patients (36.38 ng/mL) vs. controls (59.96 ng/mL).	[85]
Subclinical Hypothyroidism	160 Turkish middle-aged	No association	Similar irisin levels (23.0 vs. 22.2 ng/mL) and no link to CIMT.	[86]
CHD & Depression	209 Chinese elderly	Low levels = Risk factor	Lower in CHD with depression (158.1 pg/mL) vs. without (203.7 pg/mL).	[87]
Metabolic Syndrome (MetS)	59 Jordanian middle-aged	Contradictory/High levels in MetS	Higher in normoglycemic MetS (166.55 ng/mL) vs. controls (109.71 ng/mL).	[88]
Obesity in Children	44 Greek children	Contradictory/High levels in obesity	Higher in obese (187.96 ng/mL) vs. controls (169.73 ng/mL).	[89]
	3651 Chinese children	Context-dependent	No link to PA/ST, but negative link to BMI/DBP only in inactive children.	[90]
Overweight/Obesity & CVRFs	191 Chinese adults	High levels = Protective	High irisin group had lower prevalence of smoking, dyslipidemia, and CVRF clustering.	[91]
Genetic Polymorphisms (FNDC5 Gene)				
MI Risk (Egypt)	100 Egyptian elderly	Contradictory/Association found	G allele (rs3480) & A allele (rs726344) associated with increased MI risk.	[92]
MI Risk (Turkey)	225 Turkish elderly	Contradictory/No association	No significant genotype association for rs16835198, rs3480, rs726344.	[68]
CAD & Hypertension Risk (China)	Chinese patients	Contradictory/No association	No association found for rs16835198 and rs3480 with CAD or hypertension.	[93]
Coronary Artery Disease (CAD) & Atherosclerosis				
Stable CAD & Severity	63 Turkish elderly	Low levels = Worse outcome	High SYNTAX: 127.9 vs. Low SYNTAX: 224.7 ng/mL.	[59]
	62 Pakistani middle-aged	Low levels = Worse outcome	Lower in severe CAD (9.3 µg/mL) vs. mild CAD (15.3 µg/mL).	[60]
	45 Chinese elderly	Low levels = Risk factor	Lower in CAD (175.0 µg/L) & DELC group vs. controls.	[62]
	71 Greek patients	Low levels = Risk factor	Lower in stable CAD (161.2 ng/mL) and MI (143.5 ng/mL) vs. controls (217.2 ng/mL).	[61]
Coronary Artery Ectasia (CAE)	50 Turkish middle-aged	Low levels = Risk factor	Lower in CAE (1.85 pg/mL) vs. controls (5.78 pg/mL).	[64]
Coronary Artery Calcification (CAC)	1038 Japanese men	High levels = Protective	Higher irisin associated with lower CAC prevalence and progression.	[63]
Acute Myocardial Infarction (AMI)	225 Turkish elderly	Low levels = Risk factor	Lower in AMI (3.14 ng/mL) vs. controls (4.02 ng/mL); levels drop post-AMI.	[68]
	44 Turkish elderly (STEMI)	Low levels = Risk factor	Lower in STEMI (4.95 ng/mL) vs. controls (12.5 ng/mL). Cutoff: <6.71 ng/mL.	[69]
Aortic Stenosis & Calcification	293 Chinese elderly	Low levels = Worse outcome	Negatively associated with aortic valve calcification severity.	[65]
Obesity with Carotid Atherosclerosis	156 Chinese elderly	Low levels = Risk factor	Lower in obese CAS (28.05 µg/mL) vs. controls (63.38 µg/mL).	[67]
Subclinical Atherosclerosis (Obesity)	61 Spanish middle-aged	Low levels = Risk factor	Lower in subjects with atheroma (15.4 ng/mL) vs. without (20.9 ng/mL).	[66]
Overweight/Obesity & CVRFs	191 Chinese adults	High levels = Protective	High irisin group had lower prevalence of smoking, dyslipidemia, and CVRF clustering.	[91]
Acute Ischemic Stroke (AIS)	324 Chinese elderly	Low levels = Worse outcome	Lower in poor outcome group (257.1 ng/mL) vs. good outcome (315.5 ng/mL).	[70]
CHD & Depression	209 Chinese elderly	Low levels = Risk factor	Lower in CHD with depression (158.1 pg/mL) vs. without (203.7 pg/mL).	[87]
T2DM with Atherosclerosis	69 Egyptian middle-aged	Low levels = Risk factor	Lower in T2DM + AS (32.9 pg/mL) vs. controls (473.6 pg/mL).	[73]
T2DM & Macrovascular Complications	138 Saudi middle-aged	Low levels = Risk factor	Lower in T2DM + MVC (0.50 µg/mL) vs. without (0.58 µg/mL).	[74]
T2DM & CTO	86 Turkish elderly	Low levels = Risk factor	Lower in patients with diabetes, but not correlated with SYNTAX score.	[77]
T2DM & CVD Risk	135 Egyptian middle-aged	Low levels = Risk factor	Lower in CVD group (55.77 ng/mL) vs. non-CVD (115.5 ng/mL).	[75]
T2DM & AGEs Accumulation	362 Chinese middle-aged	Low levels = Marker of damage	Lower in T2DM (16.24 ng/mL) vs. controls (24.35 ng/mL).	[76]
Chronic HF & Function	292 Pakistani elderly	Low levels = Worse outcome	Lower in CHF (224–229 pg/dL) vs. controls; correlated with LVEF and CRP.	[83]
HF with or without MI	66 Egyptian middle-aged	Low levels = Risk factor	Lower in HF groups (48.7–54.3 ng/mL) vs. controls (73.1 ng/mL).	[79]
T2DM & HF Presence/Prognosis	226/183 Ukrainian patients	Low levels = Risk factor	Lower irisin predicts HF presence and progression to HFrEF.	[80]
Prediction of HF Improvement	313 Ukrainian elderly HFrEF	High levels = Favorable outcome	Higher in HFimpEF group (8.23 ng/mL) vs. HFrEF (4.37 ng/mL).	[81]
Axial Spondyloarthritis (axSpA)	725 Spanish middle-aged	Low levels = Risk factor	Lower in patients with sacroiliitis (2.12 ng/mL) vs. without (2.60 ng/mL).	[84]
Severe Hypothyroidism	25 Chinese middle-aged	Low levels = Marker of injury	Lower in patients (36.38 ng/mL) vs. controls (59.96 ng/mL).	[85]
Subclinical Hypothyroidism	160 Turkish middle-aged	No association	Similar irisin levels (23.0 vs. 22.2 ng/mL) and no link to CIMT.	[86]
T2DM (General)	65 Egyptian middle-aged	Context-dependent	No difference vs. controls, but inverse correlation with BMI, CRP, LDL.	[78]
Metabolic Syndrome (MetS)	59 Jordanian middle-aged	Contradictory/High levels in MetS	Higher in normoglycemic MetS (166.55 ng/mL) vs. controls (109.71 ng/mL).	[88]
Obesity in Children	44 Greek children	Contradictory/High levels in obesity	Higher in obese (187.96 ng/mL) vs. controls (169.73 ng/mL).	[89]
	3651 Chinese children	Context-dependent	No link to PA/ST, but negative link to BMI/DBP only in inactive children.	[90]
HFpEF vs. HFrEF	29 Italian elderly	Contradictory/High levels in HFpEF	Higher in HFpEF (7.72 ng/mL) vs. HFrEF (2.77 ng/mL).	[82]
Prognosis post-PCI for AMI	207 Chinese elderly	Low levels = Worse outcome	Lower in MACE group (49.35 ng/mL) vs. no-MACE (78.93 ng/mL).	[71]
Prognosis post-STEMI	399 Taiwanese middle-aged	Contradictory/High levels = Worse	Highest quartile (>0.60 μg/mL) had ~4-fold increased risk of adverse events.	[72]
MI Risk (Egypt)	100 Egyptian elderly	Contradictory/Association found	G allele (rs3480) & A allele (rs726344) associated with increased MI risk.	[92]
MI Risk (Turkey)	225 Turkish elderly	Contradictory/No association	No significant genotype association for rs16835198, rs3480, rs726344.	[68]
CAD & Hypertension Risk (China)	Chinese patients	Contradictory/No association	No association found for rs16835198 and rs3480 with CAD or hypertension.	[93]

Abbreviations: AGEs: Advanced glycation end products; AMI: Acute Myocardial Infarction; AS: Atherosclerosis; axSpA: Axial Spondyloarthritis; CAD: Coronary Artery Disease; CAE: Coronary Artery Ectasia; CAS: Carotid Atherosclerosis; CHF: Chronic Heart Failure; CIMT: Carotid Intimate Thickness; CRP: C-Reactive Protein; CTO: Chronic Total Occlusion; CV: Cardiovascular; CVD: Cardiovascular Disease; CVRFs: Cardiovascular Risk Factors; DBP: Diastolic Blood Pressure; DELC: Diagonal Earlobe Crease; HF: Heart Failure; HFrEF: Heart Failure with Reduced Ejection Fraction; HFpEF: Heart Failure with preserved Ejection Fraction; LVEF: Left Ventricular Ejection Fraction; MACE: Major Adverse Cardiovascular Events; MetS: Metabolic Syndrome; MI: Myocardial Infarction; MVC: Macrovascular Complications; PA: Physical Activity; PCI: Percutaneous Coronary Intervention; ST: Sedentary Time; STEMI: ST-Segment Elevation Myocardial Infarction; T2DM: Type 2 Diabetes Mell. Note: This table is for illustrative purposes only. The reported concentrations and cutoffs are derived from studies using markedly different assays (predominantly commercial ELISA kits of varying and often unvalidated specificity) and inconsistent units. These values are not directly comparable across studies and should not be interpreted as clinically applicable thresholds. They are presented solely to illustrate the methodological heterogeneity of the field. No validated diagnostic or prognostic threshold for circulating irisin currently exists.

**Table 3:** Illustrative comparison of reported circulating irisin concentrations and diagnostic cutoffs across studies, highlighting methodological disparities.

Clinical Context	Reported Irisin Concentration (Condition vs. Control/High vs. Low Risk)	Unit	Proposed Cutoff Value	Reference
Highest Reported Values (μg/mL)				
Obesity with Carotid Atherosclerosis	28.05 vs. 63.38	μg/mL	<34.48 μg/mL	[67]
Prognosis post-STEMI	>0.60 (High risk)	μg/mL	N/A (Quartile-based)	[71]
Mid-Range Reported Values (ng/mL)				
Obesity in Children	187.96 vs. 169.73	ng/mL	N/A (Comparison)	[89]
Stable CAD (Severity)	127.9 vs. 224.7	ng/mL	N/A (Correlation-based)	[59]
Prognosis post-PCI for AMI	49.35 vs. 78.93	ng/mL	<69.07 ng/mL	[72]
Severe Hypothyroidism	36.38 vs. 59.96	ng/mL	<36.94 ng/mL	[85]
T2DM & HF Prognosis	3.20 vs. 7.50	ng/mL	<6.50 ng/mL	[94]
Lowest Reported Values (pg/mL)				
T2DM with Atherosclerosis	32.9 vs. 473.6	pg/mL	N/A (Comparison)	[73]
Coronary Artery Ectasia	1.85 vs. 5.78	pg/mL	<2.1 pg/mL	[64]

Note: This table is illustrative of the vast range of reported values and units. The discrepancy between the highest reported values in μg/mL and the lowest in pg/mL represents a difference of more than a million-fold, underscoring the critical need for assay standardization before a definitive physiological range or clinical cutoff can be established. None of the proposed cutoff values listed here should be regarded as clinically validated or applicable in practice. "N/A" indicates that no specific numerical cutoff was proposed in the original study. In these cases, results were reported as quartile-based comparisons, simple group comparisons, or correlation analyses, without a defined diagnostic or prognostic threshold.

## Data Availability

No new data were created or analyzed in this study. Data sharing is not applicable to this article.
